# Detection of Wood Mice (*Apodemus sylvaticus*) Carrying Non-Tuberculous Mycobacteria Able to Infect Cattle and Interfere with the Diagnosis of Bovine Tuberculosis

**DOI:** 10.3390/microorganisms8030374

**Published:** 2020-03-06

**Authors:** Lucía Varela-Castro, Olalla Torrontegi, Iker A. Sevilla, Marta Barral

**Affiliations:** NEIKER-Instituto Vasco de Investigación y Desarrollo Agrario, Animal Health Department, Bizkaia Science and Technology Park 812L, 48160 Derio (Bizkaia), Spain; lvarela@neiker.eus (L.V.-C.); otorrontegui@neiker.eus (O.T.); isevilla@neiker.eus (I.A.S.)

**Keywords:** non-tuberculous mycobacteria, small mammals, *Apodemus sylvaticus*

## Abstract

Mycobacterial infections caused by the *Mycobacterium tuberculosis* complex (MTC) and non-tuberculous mycobacteria (NTM) are of great medical and veterinary relevance. The aim of this research was to study whether small mammals play a role in the epidemiology of mycobacterioses. Four samplings of 100 traps were performed in each of three cattle farms with previous history of tuberculosis or NTM between 2017 and 2018. A total of 108 animals belonging to seven species were trapped, classified, and necropsied, and tissues were submitted to microbiological and molecular methods for mycobacteria identification. The wood mouse (*Apodemus sylvaticus*) was the most abundant species (87%). No MTC was detected but six different NTM were identified (*M. intracellulare*, *M. avium* subsp. *paratuberculosis*, *M. gordonae*, *M. celatum*, *M. fortuitum,* and a not determined *Mycobacterium* sp.), showing a prevalence of 6.5%. No significant association was found between mycobacteria prevalence and the analyzed factors. Although a role in the epidemiology of MTC could not be attributed to small mammals, *A*. *sylvaticus* carries NTM that could be pathogenic or interfere with the diagnosis of tuberculosis. According to our results, there is a risk of NTM transmission at the wildlife–livestock interface through potential indirect contacts between small mammals and cattle.

## 1. Introduction

Aside from the agents responsible for leprosy, the genus *Mycobacterium* includes a large number of species that can be split into two main groups: the *Mycobacterium tuberculosis* complex (MTC) and non-tuberculous mycobacteria (NTM). Several species of mycobacteria have been detected in wild and domestic animals [1,2], in humans [3], and also in the environment, which could represent an important reservoir due to the species’ resistance to adverse factors and ubiquity [4]. Those species belonging to MTC are the most studied, since they are the causative agents of human and animal tuberculosis (TB). Human TB is a worldwide infectious disease mainly caused by *M. tuberculosis* with a 1.2 million death toll in 2018 according to the World Health Organization [5]. Animal TB is a zoonotic disease that causes severe economic losses in the livestock industry of developed countries [6]. It is mainly caused by *M. bovis*, even though other species such as *M. caprae* can be involved [7]. On the other hand, NTM are ubiquitous in a broad variety of soil and aquatic environments [8] and compose most of the species belonging to the genus *Mycobacterium*. However, because of an initial lack of knowledge on their clinical relevance, NTM were neglected for many years. Currently, conversely, they are associated with a wide range of infections in humans and animal species. Clinical manifestations caused by NTM range from skin and soft tissue infections to respiratory or digestive infections or diseases [2,8,9]. One meaningful example of veterinary relevance would be *Mycobacterium avium* subsp. *paratuberculosis (Map)*, a member of the *Mycobacterium avium* complex (MAC), which is the causative agent of paratuberculosis in ruminants. *Map* has also been related to Crohn’s disease in humans, but this still remains controversial [10]. Some species of NTM have been pointed out as a source of interference with bovine TB diagnostic reagents, such as *Map* itself, *Mycobacterium avium* subsp. *Avium,* and *Mycobacterium fortuitum* [2,11], or with the protection provided by the Bacillus Calmette Guerin (BCG) vaccination [12].

Soils shared between sympatric wildlife and livestock may become key zones for the indirect transmission of mycobacteria. Wild small mammals could have a role in the spread of these agents into those specific areas, since they are present in pastures and farm enclosures (sheds, straw, forage, etc.). Currently, rodent population control seems to be the most widespread measure to minimize the presence of small mammals within farm buildings, but the protection of forage, straw, and water to avoid small mammals feeding and excreting over these resources remains not completely solved [13]. The implementation of these measures may become even more complicated when feeders and troughs are also placed in the pastures. Pathogenic or opportunistic mycobacteria can colonize small mammals’ tissues or simply pass through their digestive system and be shed intact in feces and body fluids, which could be further spread by the movements of these animals [13]. Previous studies have described the detection of mycobacteria in small mammals. Apart from *M. microti*, *M. bovis* was isolated from urban and wild rodents [14,15] and its ability to infect different species has been experimentally demonstrated [16]. Regarding NTM detection in small mammals, *Map*, *M. intracellulare*, *M. gordonae,* and *M. chelonae* have been isolated, among others [17,18]. Other studies have simultaneously detected the same species of NTM in livestock and cohabiting small mammals or even suggested a possible transmission of mycobacteria between them [19]. Whether small mammals can act just as carriers or as true hosts or even reservoirs is not clear yet. Therefore, more in depth studies investigating the relevance of these mammals in the epidemiology of mycobacterioses are needed if we want to design effective global control strategies. The goal of this research was to study the role of small mammals in cattle farms with a history of TB or NTM, using as reference three farms located in the Basque Country, Northern Spain. We also searched for factors associated with the detection of mycobacteria in these mammals.

## 2. Materials and Methods

### 2.1. Study Area and Small Mammal Sampling

Three cattle farms from the Basque Country with history of TB and/or NTM cases (Table 1) were selected, and permissions for small mammal trapping and euthanasia were obtained from the competent authorities (corresponding approval numbers and dates: 6387/2917 in December 2017, 1907 in March 2017 and 183 in February 2017). The selected farms had reactor cattle to the intradermal tuberculin test that were subsequently confirmed as *M. bovis*-infected or as false positives. From July 2017 to October 2018, 100 traps baited with chorizo (sausage-like cured meat product) were placed for small mammal live capture at the areas where cattle were located during the sampling. The selected bait is easy to insert and remove from the traps, does not rot rapidly, and can resist harsh weather conditions and feeding by invertebrates. Traps were placed overnight once every season, making a total of four samplings per farm. Sherman traps (7.6 cm by 8.9 cm by 22.9 cm; H.B.Sherman traps Inc., Tallahassee, FL, USA) were used indoors, while INRA traps (5 cm by 5 cm by 15 cm; BTS Mechanique, Besançon, France) were used along the edges between pastures and adjacent forests’ shrubs.

### 2.2. Processing of Small Mammals and Sample Preparation

**Figure 1:**
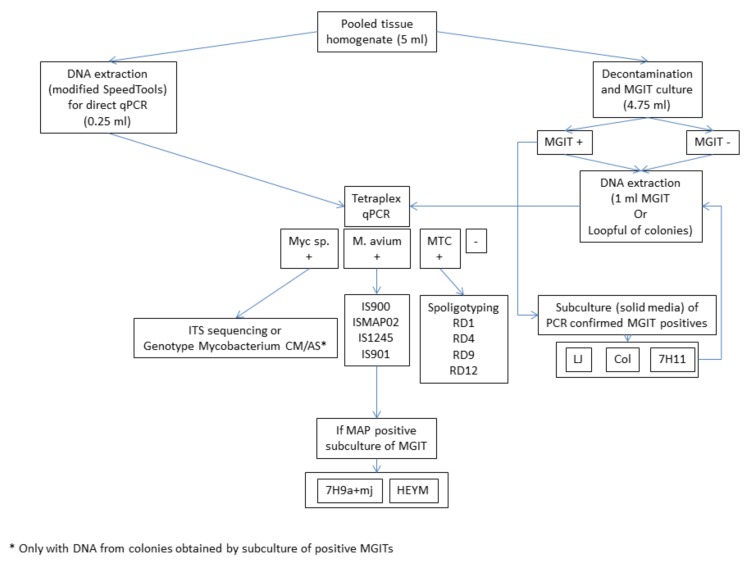
Schematic representation of the methodology. Culture media abbreviations: MGIT = Mycobacteria Growth Indicator Tube; LJ = Löwenstein–Jensen; Col = Coletsos; 7H11 = Middlebrook 7H11 supplemented with oleic acid-albumin-dextrose-catalase (OADC) enrichment; 7H9a+mj = agar-solidified 7H9 medium supplemented with OADC and mycobactin J; HEYM = in-house Herrold´s Egg Yolk medium containing sodium pyruvate and mycobactin J. + = positive result; - = negative result.

Captured individuals were transported to a Biosafety level 3 Laboratory and euthanized in a CO_2_ chamber. Afterwards, weight and biometrics of each individual were recorded. At necropsy, sex and age (adult or juvenile) were determined and organs were inspected for the presence of macroscopic lesions. A pool of tissues was prepared for each animal including lymph nodes from the head, the respiratory system, and the intestinal tract, lung, ileum, and muscle. All pools weighed less than 1 g. Finally, small mammal species were identified by dental alveoli patterns and skull and biometric features, following the indications of taxonomic keys and morphological studies [20,21]. Prior to further processing, tissue pools were homogenized in 5 mL of sterile distilled water using a GentleMACS™ Dissociator (Miltenyi Biotec, Madrid, Spain) (RNA_02 program) and divided into two aliquots of 4.75 mL and 0.25 mL for culture and direct DNA extraction and real-time PCR analysis, respectively. A schematic representation of the laboratory methodology is given in Figure 1.

### 2.3. Culture

Considering the small size of samples (<1 g), almost the whole volume of homogenized sample (4.75 mL) was destined to a single culture procedure. Homogenates were decontaminated using the BD BBL™ MycoPrep™ kit and processed for culture in BBL™ mycobacteria growth indicator tubes (MGIT™) (Becton Dickinson, Franklin Lakes, NJ, USA) supplemented with BACTEC™ MGIT™ growth supplement and PANTA™ antibiotic mixture according to the manufacturer’s instructions (Becton Dickinson, Franklin Lakes, NJ, USA). Inoculated MGITs were incubated in an automated BACTEC MGIT 960 system (Becton, Dickinson and Company, Sparks, MD, USA) at 37 °C for an extended period of at least four months to enable isolation of slowly growing mycobacteria.

MGIT cultures confirmed as positive were subcultured in Difco Löwenstein–Jensen, Coletsos (Dismalab S.L., Madrid, Spain), and Middlebrook 7H11 supplemented with oleic acid-albumin-dextrose-catalase (OADC) enrichment (Becton Dickinson, Franklin Lakes, NJ, USA) in order to obtain isolated colonies for further molecular characterization. Since *Map* needs exogenous addition of mycobactin J for in vitro culture [22], its growth requirements were not covered by the culture medium chosen in this study for primary isolation. To circumvent this methodological bias, if a DNA sample tested PCR-positive for *Map*, regardless of being DNA extracted from tissue homogenate or MGIT culture, its corresponding MGIT was subcultured in in-house prepared Herrold´s Egg Yolk medium (HEYM) containing sodium pyruvate and mycobactin J (IDvet, Grabels, France) and in agar-solidified 7H9 medium supplemented with OADC and mycobactin J.

### 2.4. DNA Extraction

DNA extraction from tissue homogenate aliquots (0.25 mL) was performed using a modified protocol of the Speedtools Tissue DNA extraction kit (BioTools, B&M Labs S. A., Madrid, Spain) as described previously [23,24]. DNA was extracted from all MGIT cultures regardless of having positive or negative BACTEC time to detection (TTD) readouts. One milliliter of MGIT culture was centrifuged at 16,000× *g* for three minutes and the supernatant discarded. Pellets were resuspended in 0.25 mL of distilled water, inactivated at 90 °C for 20 min, and submitted to DNA extraction using the same modified protocol specified above for tissue homogenates.

### 2.5. Tetraplex Real-Time PCR for the Screening of Tissues and Cultures

A previously described [23] and modified [24] tetraplex real-time PCR was performed for the screening of DNA extracted from MGIT cultures and homogenized tissue pools. This technique allows for the simultaneous detection of the *Mycobacterium* genus, all four *M*. *avium* subspecies, and MTC. The reaction was carried out in a total volume of 25 µL, containing 3 µL of extracted DNA and 22 µL of mastermix. Amplification was carried out in a 7500 Real-Time PCR thermal cycler (Applied Biosystems, Foster City, CA, USA) under previously described conditions [23,24]. The estimation of valid cycle threshold (CT) and baseline was calculated automatically with the SDS software v. 1.5.1 (Applied Biosystems, Foster City, CA, USA), visually confirmed by checking amplification plots, and manually adjusted if needed.

### 2.6. Further Molecular Identification of Mycobacteria Detected by the Tetraplex Real-Time PCR

#### 2.6.1. Identification of *Mycobacterium* sp.-Positive Samples

*Mycobacterium* sp. detected by the tetraplex real-time PCR of DNA samples extracted from tissue homogenates were further identified by PCR and sequence analysis of the 16S-23S rRNA internal transcribed spacer (ITS). A previously described nested PCR was used for PCR amplification of the ITS region [25]. After electrophoresis, PCR products were purified from agarose gels with the Genelute Gel Extraction kit (Sigma-Aldrich Co. Ltd., St. Louis, MO, USA) as recommended by the manufacturer. Purified amplicons and the same primers used for the second round of the nested PCR were adjusted to appropriate concentrations and shipped to EuroFins GATC Biotech GmbH (Konstanz, Germany) for sequencing. Inspection, edition, and alignment of sequences was performed, assisted by Sequencing Analysis 5.2 software (Applied Biosystems, Foster City, CA, USA), and then compared with other published sequences using online BLAST analysis (NCBI, NLM, Bethesda, MD, USA).

*Mycobacterium* sp. isolates were identified using the Genotype Mycobacterium CM and AS kits (Hain Lifesciences GmbH, Nehren, Germany). For this purpose, a loopful of colonies growing in solid subcultures was resuspended in 100 µl of A-LYS/IC reagent of the GenoLyse kit (Hain Lifesciences GmbH, Nehren, Germany), and DNA was extracted following the protocol provided with the kit. Then, DNA was amplified and PCR amplicon identity revealed using the Genotype Mycobacterium CM and AS kits and the Twincubator hybridizer (Hain Lifesciences GmbH, Nehren, Germany) according to the indications of the manufacturer. These kits contain membrane strips coated with specific probes that are complementary to certain mycobacterial DNA sequences, allowing for the identification of MTC and 27 species of NTM in agreement with the hybridization pattern obtained. Isolates that could not be identified at the species level with this kit were further identified by the aforementioned ITS sequencing procedure.

#### 2.6.2. Identification of *M. avium* subsp.-Positive Samples

For subspecies identification of samples yielding a positive result for *M*. *avium* in the tetraplex real-time PCR, DNA was analyzed by different real-time or conventional PCR methods described earlier to amplify IS*900*, IS*Map02* [26], IS*1245,* and IS*901* [27]. Identification was enabled by the interpretation of presence–absence signatures obtained for the genomic targets interrogated by PCR, which are subspecies-specific [24]: *Map* is IS*900*+, IS*Map02*+, IS*1245*−, IS*901*−; *M. avium* subsp. *avium* (and subsp. *silvaticum*) is IS*900*−, IS*Map02*−, IS*1245*+, IS*901*+; *M. avium* subsp. *hominissuis* is IS*900*−, IS*Map02*−, IS*1245*+, IS*901*−.

### 2.7. Identification of MTC-Positive Samples

The strategy outlined for the identification of MTC-positive samples included standard spoligotyping [28] as well as amplification of the regions of difference (RD) 1, 4, 9, and 12 of *M*. *tuberculosis* using previously described primers [29] in independent conventional singleplex PCR assays [24]. The RD signature patterns for MTC species identification have been specified earlier [29].

### 2.8. Statistical Analyses

Mycobacteria detection (positive/negative) and factors such as animal age, sex, season of capture, and sampling locality were analyzed using Fisher´s test. The combined results of direct PCR and culture were used as the dependent variable. Significance was set at *p* < 0.05. Statistical analyses were performed using the R Software 3.5.0 (R Development Core Team, 2018, Vienna, Austria).

## 3. Results

### 3.1. Identification and Processing of Small Mammals

A total of 108 small mammals, 50 females (29 adults and 21 juveniles) and 58 males (28 adults, 25 juveniles and five undetermined), were trapped. Six species of rodents and one shrew species were identified, with *Apodemus sylvaticus* being the most frequently trapped species (87%; see Table 1 for further details). One rodent belonging to *Apodemus* genus could not be further identified due to massive teeth wear. Two individuals showed macroscopic lesions in the liver and in the kidney, respectively, but were not compatible with mycobacterial infections as assessed through histopathological and microbiological analyses.

### 3.2. Mycobacteria Detection and Identification

No members of the MTC were detected. As for NTM, the overall prevalence was 6.5% (7/108; 95% CI, (3.2–12.8%)). More specifically, three species belonged to *M. avium* subspecies and four belonged to other NTM. Among them, one was detected in the unidentified *Apodemus* specimen and the other six were detected in *A*. *sylvaticus* individuals (Table 2). However, no animal tested positive for both direct PCR and culture.

Out of the 89 MGIT cultures displaying a positive TTD readout, only four were confirmed to contain mycobacteria with the tetraplex real-time PCR. The identification of these four isolates with the Genotype Mycobacterium CM and AS reverse hybridization kits was as follows: *M. fortuitum* complex (the ITS sequence obtained for this isolate displayed a percentage of identity of 98.74% with *Mycobacterium sp*. DL90, 96.68% with *M. fortuitum* sequevar Mfo D 16S-23S, and 96.68% with *M. fortuitum* strain S358 in BLAST analysis), *M. intracellulare*, *M. gordonae,* and *M. celatum* (Table 2).

As for the homogenized tissue pools, two were positive to *M*. *avium* subspecies and one was positive to other NTM, according to the tetraplex real-time PCR. The sequence obtained for the sample positive to *Mycobacterium* sp. best matched with the ITS sequence available in GenBank for *M*. *peregrinum* isolate IoA5, displaying a percentage of identity of 82.91%, according to BLAST analysis. The two *M. avium* subspecies detected in the tissues of two animals by the tetraplex PCR were identified as *Map,* in agreement with the insertion sequence signature obtained (IS*900*+, IS*Map02*+, IS*1245*− and IS*901*).

### 3.3. Statistics

Statistical analyses were performed considering only those individuals belonging to well-represented animal species, in this case, only *A*. *sylvaticus*. No statistically significant differences were detected in NTM distribution according to sex or age of small mammals, season, or farm (Table 3).

## 4. Discussion

Studies researching the potential role of small mammals in the epidemiology of mycobacterial infections are lacking. To the best of our knowledge, this is the first reported survey searching for mycobacteria in small mammals present in Spanish cattle farms with a history of mycobacterioses. Most of the mycobacteria detected in this study were only found in *A*. *sylvaticus*. This species was also the most frequently trapped (Table 1) and it is the most abundant within the forests of the Iberian Peninsula, inhabiting a wide range of habitats [30]. The type of traps and bait used in this study could have had a negative impact on targeting some species that are mainly herbivores (*Microtus gerbei, Microtus agrestis,* and *Myodes glareolus*).

Out of the 89 MGIT cultures displaying a positive TTD readout, 85 were not confirmed with the tetraplex real-time PCR. This high proportion of contaminated MGITs suggests that an improved decontamination and culture protocol for this type of samples might have yielded more mycobacterial isolates, since mycobacterial culture is generally problematic and very sample-matrix specific in terms of the procedure adopted. With regard to the positive individuals, direct PCR and culture results were discrepant in all the cases. For those samples displaying a positive direct PCR but a negative culture, one explanation could be that while culture will only recover live mycobacteria, PCR can detect the DNA of both live and dead cells. Samples with positive direct PCR displayed high CTs (CTs > 35), which are normally related to a low bacterial load. In addition to this, the MGIT cultures of these samples were all contaminated, and thus, mycobacterial growth could have been prevented by the growth of contaminating flora. Regarding the two samples that were *Map*-positive by direct PCR, it is clear that MGIT without mycobactin J is not a suitable medium for *Map*. In spite of the attempt to recover *Map* cells by subculturing the MGIT broth of both samples in HEYM and M7H11, we were not able to isolate any *Map* colonies. On the other hand, those cases showing a positive culture but a negative direct PCR could be attributed to a higher sensitivity of culture over PCR. According to previous results, the minimum detectable concentration of *M*. *kansasii* (acting as a proxy for non-*M*. *avium* NTM) in artificially inoculated samples can be one CFU log unit lower for MGIT-BACTEC than for the unmodified protocol of the tetraplex real-time PCR employed [23]. It should also be mentioned that the volume of homogenized sample used for culture was almost 20 times the volume used for DNA extraction, from which only 3 out of 100 µl of the eluted DNA were loaded per PCR reaction.

Although we did not detect any species belonging to the MTC, we did find other mycobacteria of interest. For instance, two members of MAC were detected, *M. intracellulare* and *Map*, which have been demonstrated to sensitize cattle and interfere in the diagnosis of TB [8,11]. *M. intracellulare* is a NTM commonly found in patients with mycobacterial pulmonary disease [9]. It has been recovered from water, soil, and biofilm samples [31] and it is also implicated in infections of several wild and domestic animals, including cattle [32]. As for small mammals, this bacterium was previously detected in the lungs of African rodents and insectivores [17]. *Map* is the causative agent of paratuberculosis, a chronic wasting disease that mainly affects ruminants, even though it has been isolated from many other wild and domestic species [8]. Although some rodents seem to be resistant to *Map* infection [33], this bacterium has been previously detected in *A. sylvaticus* [34]. On the other hand, *M. fortuitum*, *M. gordonae, M. celatum,* and a not determined *Mycobacterium* sp. with an ITS sequence similar to *M. peregrinum* (83% sequence identity) were also detected in this study. *M. fortuitum* is related to lung disease in humans and has been related to immune sensitization in cattle, leading to cross-reactive responses that can interfere with the diagnosis of tuberculosis [35]. This species has been described as naturally pathogenic for mice [36], and it has previously been detected in *Microtus arvalis* [13]*. M. gordonae* is the most commonly isolated mycobacterial species due to contamination when human respiratory specimens are cultured, even though it also can cause pulmonary or disseminated infection [9]. It has also been detected in cattle [37] and several species of small mammals [17]. This bacterium has been commonly found in water reservoirs [38]. Besides, it has been described as an NTM species that could potentially express cross-reactive antigens and, consequently, affect the tuberculin test specificity [39]. *M. celatum* is an infrequently detected species of *Mycobacterium,* which is more common among immunocompromised patients [32]. Its detection in animals is even less frequent and only two cases of *M. celatum* infection in domestic ferret (*Mustela putorius furo*) and one in a white-tailed trogon (*Trogon viridis*) have been reported [40]. Even though mice can be susceptible to *M. celatum* during experimental infection [41], this is the first report on the detection of this species in free-living rodents. It is worth mentioning that *M. celatum* can cause false *M. tuberculosis*-positive results in commercial molecular identification tests [9]. Lastly, *M. peregrinum* has been described as an opportunistic pathogen for humans and livestock [2], even though it has also been detected in wild animals [42].

Despite these findings, it was not possible to discern between true infection and passing-through microorganisms. Even though the small size of these animals may hinder the detection of macroscopic lesions, no visible lesions consistent with mycobacterial infection were observed in positive or negative animals. Pooling of tissues implies a loss of information about the body site where mycobacteria were located, and thus, possible entrance or excretion routes could not be further investigated. However, we have demonstrated that at least one small mammal species, *A*. *sylvaticus*, can act as a carrier of several NTM, among which *Map* is the only mycobacteria previously found in cattle from the same farm. Although *A*. *sylvaticus* does not seem to play a relevant role in the epidemiology of the MTC in our study area, we cannot reject the competence of this species to carry MTC if we take into account its ability to carry other mycobacteria, such as the NTM that have been isolated. The scarce information available in the literature on the epidemiology of natural *M*. *bovis* infection in small mammals does not suggest that *Apodemus* sp. or other small mammal species could maintain the infection in their population, and may be better considered as dead-end hosts [14,15,43]. Besides, TB prevalence was minimal during the study period among cattle and wildlife from the Basque Country, and, consequently, it is not striking that *M. bovis* was not found in small mammals. In contrast, the field vole (*Microtus agrestis*) is considered the maintenance host for *M*. *microti*, a role that similarly might be played by other small mammals [44,45]. This MTC member seems to be more widespread and infecting more species than previously thought, and has been recently identified in domestic and wild populations in France and northeastern Spain [46,47,48]. We did not detect *M*. *microti*, but it could also be that its prevalence is not high enough to favor the detection of infected animals, considering the limitations of our sample size and spatial scale. We cannot rule out the presence of mycobacteria in other small mammal species either, since the number of trapped individuals was very low.

Owing to the small representation of species other than *A. sylvaticus*, we focused the statistical analyses only towards this species. However, no statistically significant differences were detected in mycobacteria prevalence according to the analyzed variables, albeit some tendencies were visualized. For instance, a higher prevalence was observed in females. Males normally have a bigger home range, particularly during the reproductive period [30], that could give them more chances to come in contact with mycobacteria. Nevertheless, females can be subjected to a higher reproductive stress and opportunistic infections could be more effective in immunocompromised individuals. On the other hand, juveniles showed a higher prevalence as compared to that of the adults. Accumulated risk of infection with increasing age has been previously described for *M. bovis* in other wild species, such as the European badger (*Meles meles*) [49]. The same pattern could have been expected with other mycobacteria but, conversely, we have found the opposite situation. As for seasonality, both winter and summer showed a higher prevalence than autumn and spring. The small number of animals trapped during summer may have boosted the observed prevalence in this study, since only nine individuals were captured during this season. During winter, limited food sources could have promoted the entrance of small mammals into the farm buildings, increasing contact with cattle and, thus, mycobacteria circulation and exposure. When analyzing the effect of location, individuals trapped in one of the farms were all negative (Table 1). This could be related to the previous health status of this farm, which presented several outbreaks of paratuberculosis and tuberculosis, leading to a stronger implementation of biosecurity and sanitary measures that could have decreased the environmental load of mycobacteria. Despite the absence of a strong statistical relation between NTM prevalence and the explored variables in this study, if small mammals are attributed a vector role, their loitering behavior could pose a risk of uncontrolled dissemination of mycobacteria or other agents of veterinary relevance among farms and their surroundings. 

This is the first report on the detection of mycobacteria in small mammals captured in cattle farms from Spain and the first description of *M. celatum* detection in wild rodents. Conclusively, our results indicate that small mammals such as *A*. *sylvaticus* can carry potentially pathogenic NTM with the ability to cross-react with TB diagnosis in cattle, but do not seem to play a role in the epidemiology of TB in our study area and period. Due to the indirect interactions between small mammals and cattle that may take place in the environment of farms, a risk of mycobacteria transmission cannot be ruled out. Hence, further studies are required to determine the actual role of small mammals in the epidemiology of mycobacterial infections, as well as to assess if other species of small mammals are implicated. In line with this, active surveillance of NTM in cattle should be promoted in order to delve into the epidemiology of these bacteria at the wildlife–livestock interface. In addition, novel biosecurity measures directed at minimizing the likelihood of contact between livestock and small mammals should be studied and implemented in agreement with the results obtained in further research.

## Figures and Tables

**Table 1:** Mycobacteria detected in cattle and small mammals at farm level.

Farm Locality	*Mycobacterium* Species Identified in Cattle (2014–2017)	Small Mammal Species (N)	Total Number Trapped	Mycobacteria Prevalence (%) in Small Mammals (95% CI)	*Mycobacterium* Species Identified in Small Mammals
Deba			34	11.8 (4.7–26.6)	
	*M. bovis*	*Apodemus sylvaticus* (29)			*M. intracellulare*
	*Map*	*Mus domesticus* (2)			*Map*
		*Microtus agrestis* (1)			*M. fortuitum*
		*Microtus gerbei* (1)			*M. gordonae*
		*Apodemus* sp. (1)			
					
					
Kortezubi			34	8.8 (3.0–23.0)	
	*M. avium* subsp. *avium*	*A. sylvaticus* (32)			*Map*
		*Crocidura russula* (2)			*Mycobacterium* sp.^¥^
					*M. celatum*
					
					
					
					
Kexaa			40	0.0 (0.0–8.7)	
	*M. bovis*	*A. sylvaticus* (33)			
	*Map*	*Apodemus flavicollis* (3)			
	*M. avium* subsp. *avium*	*Myodes glareolus* (1)			
	*Mycobacterium* sp.*	*M. gerbei* (1)			
		*M. domesticus* (1)			
		*C. russula* (1)			

* internal transcribed spacer (ITS) sequence showing 71–75% base identities with the ITS sequence of different isolates of *M*. *insubricum* in BLAST analysis. ^¥^ The sequenced ITS amplicon showed a percentage of identity of 82.91% with *M*. *peregrinum* (BLAST). N = number of trapped animals.

**Table 2:** Mycobacteria detection and identification in positive small mammal specimens.

Rodent Species	*Mycobacterium* Isolation	MGIT PCR Result	Direct PCR Result (Tissue Homogenate)	*Mycobacterium* Identification Method	Final Identification
*A. sylvaticus*	Yes	Positive (*Mycobacterium* sp.)	Negative	Reverse hybridization and ITS sequencing	*M. fortuitum*
*Apodemus* sp.	Yes	Positive (*Mycobacterium* sp.)	Negative	Reverse hybridization	*M. intracellulare*
*A. sylvaticus*	Yes	Positive (*Mycobacterium* sp.)	Negative	Reverse hybridization	*M. gordonae*
*A. sylvaticus*	Yes	Positive (*Mycobacterium* sp.)	Negative	Reverse hybridization	*M. celatum*
*A. sylvaticus*	No	Negative	Positive (*M*. *avium*)	IS*900*, IS*Map02*, IS*1245* and IS*901*	*Map*
*A. sylvaticus*	No	Negative	Positive (*M*. *avium*)	IS*900*, IS*Map02*, IS*1245* and IS*901*	*Map*
*A. sylvaticus*	No	Negative	Positive (*Mycobacterium* sp.)	ITS sequencing	*Mycobacterium* sp.*

* ITS sequence with a percentage of identity of 82.91% with *M*. *peregrinum* IoA5 (BLAST).

**Table 3:** Prevalence of non-tuberculous mycobacteria (NTM) detected in *A. sylvaticus* according to the categorical variables.

Variable	Number Tested	% Positives (95% CI)	*p* Value
**Sex**			1
Female	42	7.1 (2.5–19.0)	
Male	52	5.8 (2.0–15.6)	
**Age**			1
Juvenile	40	7.5 (2.6–19.9)	
Adult	50	6.0 (2.1–16.2)	
**Season**			0.3
Autumn	23	0.0 (0.0–14.3)	
Winter	36	11.1 (4.4–25.3)	
Spring	26	3.8 (0.7–18.9)	
Summer	9	11.1 (2.0–43.4)	
**Farm Locality**			0.1
Deba	29	10.3 (3.6–26.4)	
Kortezubi	32	9.4 (3.2–24.2)	
Kexaa	33	0.0 (0.0–10.4)	
